# Therapeutic effects of CXCR4^+^ subpopulation of transgene‐free induced cardiosphere‐derived cells on experimental myocardial infarction

**DOI:** 10.1111/cpr.13041

**Published:** 2021-05-04

**Authors:** Jianyong Xu, Huimei Wu, Zhigang Mai, Junbo Yi, Xianqi Wang, Lingyun Li, Zhong Huang

**Affiliations:** ^1^ Guangdong Provincial Key Laboratory of Regional Immunity and Diseases Department of Immunology Health Science Center Shenzhen University Shenzhen China

**Keywords:** CXCR4, induced cardiosphere, intravenous transplantation, myocardial infarction, transgene‐free

## Abstract

**Objectives:**

Myocardial infarction (MI) is the most predominant type of cardiovascular diseases with high mortality and morbidity. Stem cell therapy, especially cardiac progenitor cell therapy, has been proposed as a promising approach for cardiac regeneration and MI treatment. Previously, we have successfully generated cardiac progenitor‐like cells, induced cardiosphere (iCS), via somatic reprogramming. However, the genome integration characteristic of virus‐based reprogramming approach hampered their therapeutic applications due to the risk of tumour formation. In the current study, we aim to establish a safer iCS generation strategy with transgene‐free approaches.

**Materials and Methods:**

Four transgene‐free approaches for somatic reprogramming, including episome, minicircle, self‐replicative RNA, and sendai virus, were compared, from the perspective of cardiac progenitor marker expression, iCS formation, and cardiac differentiation. The therapeutic effects were assessed in the mouse model of MI, from the perspective of survival rate, cardiac function, and structural alterations.

**Results:**

The self‐replicative RNA approach produced more iCS, which had cardiomyocyte differentiation ability and therapeutic effects on the mouse model of MI with comparable levels with endogenous cardiospheres and iCS generated with retrovirus. In addition, the CXCR4 (C‐X‐C chemokine receptor 4) positive subpopulation of iCS derived cells (iCSDC) delivered by intravenous injection was found to have similar therapeutic effects with intramyocardial injection on the mouse model of MI, representing a safer delivery approach.

**Conclusion:**

Thus, the optimized strategy for iCS generation is safer and has more therapeutic potentials.

## Graphical abstract

Previously, we and others have successfully reprogrammed mouse fibroblast cells into cardiac progenitor‐like cells. However, several issues should be further addressed, including the reprogramming strategy and delivery approach for myocardial infarction (MI) treatment. In the current study, we screened several transgene‐free approaches for somatic reprogramming. Our data showed the self‐replicative RNA approach produced more iCS, which had cardiomyocyte differentiation ability and therapeutic effects on the mouse model of MI with comparable levels with endogenous cardiospheres and iCS generated with retrovirus. Then, the CXCR4^+^ (C‐X‐C chemokine receptor 4) subpopulation of iCS derived cells (iCSDC) delivered by intravenous injection was found to have similar therapeutic effects with intramyocardial injection on the mouse model of MI, representing a more safer delivery approach. Thus, the optimized strategy for iCS generation is much safer and has more therapeutic potentials.

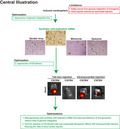

## 1 INTRODUCTION

Myocardial infarction (MI) is the most predominant type of cardiovascular diseases with high mortality and morbidity.^1^, ^2^, ^3^ Despite the available therapies have significantly improved the survival of the patients with MI, the progressive cardiac remodelling induced by MI eventually leads to heart failure and death.^4^ Stem cell therapy, especially cardiac progenitor cell therapy had been proposed as a promising approach for cardiac regeneration and MI treatment.^5^, ^6^ Unfortunately, recently it has been noticed that the endogenous cardiac progenitor cells might not exist in the adult heart.^7^, ^8^ Thus, alternative strategies should be developed to promote cardiac regeneration after MI.

Previously, we and others had successfully converted mouse fibroblast cells into cardiac progenitor‐like cells via somatic reprogramming.^9^, ^10^, ^11^, ^12^ And our previous investigations showed that the induced cardiosphere (iCS), one type of cardiac progenitor cells, had the ability to differentiate into cardiomyocytes in vitro and in vivo.^10^ And these induced cardiospheres also possess comparable therapeutic effects with endogenous cardiospheres in a mouse model of MI.^10^ However, several issues should be further addressed, including the reprogramming strategy and delivery approach for MI treatment. Firstly, the genome integration characteristic of retrovirus/lentivirus hampered their therapeutic applications due to the risk of tumour formation. Secondly, the intramyocardial injection would introduce extra injuries to the heart, although this approach shows better engraftment and therapeutic effects in the animal models.^10^, ^13^

CXCL12‐CXCR4 (C‐X‐C chemokine ligand 12/C‐X‐C chemokine receptor 4) pathway contributes to tissue regeneration via recruiting stem cells to the injured sites,^14^ and thus has been applied to improve the efficacy of stem cell therapy on MI.^15^, ^16^, ^17^, ^18^, ^19^ In the current study, we screened several transgene‐free approaches for somatic reprogramming, including episome,^20^ minicircle,^21^ self‐replicative RNA^22^ and sendai virus.^23^ Our data showed the self‐replicative RNA approach produced more iCS, which had cardiomyocyte differentiation ability and therapeutic effects on the mouse model of MI with comparable levels with endogenous cardiospheres and iCS generated with retrovirus. Then, the CXCR4^+^ subpopulation of iCS derived cells (iCSDC) delivered by intravenous injection was found to have similar therapeutic effects with intramyocardial injection on the mouse model of MI, representing a safer delivery approach. Thus, the optimized strategy for iCS generation is safer and has more therapeutic potentials.

## 2 MATERIALS AND METHODS

### 2.1 Cell isolation and maintenance

Mouse fibroblast cells were isolated and maintained as described before.^10^ Briefly, one small piece ear was removed from a 4‐week‐old mouse. Then, the tissues were disinfected with 70% ethanol, washed twice with PBS containing 200 unit/mL penicillin and streptomycin (Gibco), and incubated in digestion buffer (4 mg/mL collagenase B and 4 mg/mL dispase in DMEM/high Glucose) for 30 minutes at 37°C. Fibroblast cells were expanded in DMEM/high Glucose plus 10% FBS (foetal bovine serum, Gibco). Cells in passage 3 were used for subsequent experiments. Endogenous cardiosphere, neonatal and adult cardiomyocytes were isolated as described before.^10^ For endogenous cardiospheres (eCS) isolation, the mice (4‐week‐old) were anaesthetized with ketamine (100 mg/kg) plus xylazine (10 mg/kg). The heart was removed and washed with PBS (Gibco) via aorta perfusion, followed by digestion buffer perfusion (4 mg/mL collagenase B plus 4 mg/mL dispase in DMEM/High Glucose) at 37°C for 30 minutes. Single cells were washed twice with PBS and expanded with expanding medium (DMEM/High Glucose, 10% FBS, 100 U/mL penicillin, 100 μg/mL streptomycin, 2 mmol/L L‐glutamine and 0.1 mmol/L 2‐mercaptoethanol). Four weeks later, cells were plated onto poly‐d‐lysine (Sigma) coated dishes with CS medium (35% complete IMDM/65% DMEM‐Ham F‐12 mix containing 2% B27, 0.1 mmol/L 2‐mercaptoethanol, 10 ng/mL epidermal growth factor (EGF), 20 ng/mL basic fibroblast growth factor (bFGF), 40 nmol/L cardiotrophin‐1, 40 nmol/L thrombin, 100 U/mL penicillin, 100 µg/mL streptomycin and 2 mmol/L L‐glutamine). The eCS appear around three days after plating.

### 2.2 Induced cardiosphere generation and differentiation

The generation of iCS and differentiation was performed as described before.^10^ The retroviruses expressing Oct4, Sox2 and Klf4 were prepared and delivered into fibroblast cells according to the published procedures.^10^ The preparation of vectors overexpressing Oct4, Sox2 and Klf4 with episome, minicircle and synthetic self‐replicative RNA was performed as described before.^20^, ^21^, ^22^ The Sendai virus was purchased from Invitrogen (CytoTune™‐iPS 2.0 Sendai Reprogramming Kit). For minicircle approach, the mouse fibroblast cells were nucleotransfected at day 0, followed by another two rounds of transfection at day 4 and 6. For episome, self‐replicative RNA and sendai virus approach, the cells were nucleotransfected/transfected/infected only once at day 0. For cardiomyocytes differentiation, the iCS were plated onto Matrigel (1:40, BD Biosciences), and maintained with cardiac differentiation medium (DMEM/High Glucose, 15% FBS (HyClone), 2 mmol/L GlutaMAX, 1% nonessential amino acids, 0.1 mmol/L 2‐mercaptoethanol (Gibco), 50 μg/mL Ascorbic acid (Sigma)) for 15 days.

### 2.3 RNA extraction and real‐time PCR

Total RNAs were purified with TRIzol Reagent (Ambion) and converted into cDNA with MMLV Reverse Transcriptase (NEB) plus random primers (Invitrogen). And quantitative real‐time PCR was performed as described before.^10^

### 2.4 Immunofluorescence

Cells were plated and treated as described before.^10^ Primary antibodies include Mesp1 (Abcam, 1:100, Cat. No. ab129387), Isl1 (Abcam, 1:100, Cat. No. ab86472), Nkx2.5 (Santa Cruz, 1:100, Cat. No. sc‐376565), α‐Actinin (Sigma, 1:1000, Cat. No. A7811), myosin light chain 2 atrial (Mlc2a, Abcam, 1:100, Cat. No. ab137063) and myosin light chain 2 ventricular (Mlc2v, AXXORA, 1:10, Cat. No. ALX‐BC‐1150‐S‐L001). Coverslips were washed and then incubated with secondary antibody Alexa Fluor^®^ 488 conjugate for 1 hour. For counting Mesp1, Isl1 and Nkx2.5 positive cells, 10 random views per coverslip were counted and 5 coverslips were used for each round of experiment. The experiment was repeated three times.

### 2.5 Flow cytometry and cell purification

Flow cytometry was performed as described before.^10^ Primary antibodies include α‐Actinin (Sigma, 1:1000, Cat. No. A7811), myosin light chain 2 atrial (Mlc2a, Abcam, 1:100, Cat. No. ab137063), myosin light chain 2 ventricular (Mlc2v, AXXORA, 1:10, Cat. No. ALX‐BC‐1150‐S‐L001), c‐Kit (Abcam, 1:100, Cat. No. ab25022), Flk1 (Abcam, 1:100, Cat. No. ab233693) and CXCR4 (Abcam, 1:100, Cat. No. ab181020). The CXCR4^+^ iCSDC subpopulation was purified by BD FACSAria SORP cell sorter (BD Biosciences).

### 2.6 Cardiomyocytes characterization

The whole‐cell patch clamp and calcium transient analysis were conducted as described before.^10^ Cardiomyocytes were exposed to 10 mmol/L caffeine or 10 μmol/L beta‐adrenergic agonist Isoproterenol.

### 2.7 Mouse model of MI and heart function assessment

Mice were anaesthetized with ketamine (100 mg/kg) plus xylazine (10 mg/kg) via intraperitoneal injection 15 minutes before the surgery. Mouse model of MI and intramyocardial cell transplantation were performed as before.^10^ For intravenous injection, iCSDC was resuspended in 0.2 mL PBS and delivered via tail‐vein injection. Engineered sheet containing iCS and extracellular matrix was constructed as described before.^24^, ^25^ Briefly, the rat placenta was perfused with PBS for 15 minutes, followed by 1%SDS for 12 hours, ddH_2_O for 30 minutes, 1%Triton X‐100 for 3 hours, and finally PBS with 100 unit/mL penicillin and streptomycin for 5 days with refreshing every 12 hours. Then, the iCS was seeded onto the decellularized extracellular matrix for 12 hours followed by transplantation. The heart function was assessed by echocardiography and hemodynamic assessment as described before.^10^ The infarction size was evaluated with trichrome staining (ScyTek) as described before.^10^ The mouse studies were approved by the Animal Research Ethics Committee of Shenzhen University and conformed to the guidelines from the NIH Guide for the Care and Use of Laboratory Animals.

### 2.8 DiR labelling and cell tracking

Cells were labelled as described before.^10^ Briefly, single cells were labelled with DiR (Life Technologies) at 37°C for 15 minutes, followed by three washes with PBS. Labelled cells were transplanted into MI mice. Four weeks after cell transplantation, the heart, lung, liver, spleen and kidney were dissected out for signal detection with the IVIS Spectrum Preclinical In Vivo Imaging System (PerkinElmer). The quantity of the signals was analysed by IVIS Spectrum software.

For measuring the cell survival rate after transplantation, single cells were infected with lentivirus expressing GFP before transplantation. Four weeks after transplantation, the heart was dissected and cells were isolated with the Langendorff retrograde perfusion method.^10^ Briefly, 200 IU/mouse heparin was injected intraperitoneally 15 minutes before the mice were sacrificed. The heart was carefully dissected out and transferred to cold perfusion buffer (113 mmol/L NaCl, 4.7 mmol/L KCl, 0.6 mmol/L KH_2_PO_4_, 0.6 mmol/L Na_2_HPO_4_, 1.2 mmol/L MgSO_4_, 10 mmol/L Na‐HEPES, 12 mmol/L NaHCO_3_, 10 mmol/L KHCO_3_ and 5.5 mmol/L glucose. pH 7.0). The aorta was identified and ligated to the cannula of the Langendorff retrograde perfusion system. The heart was perfused with perfusion buffer first and then digestion buffer (400 U/mL type II collagenase (Worthington Biochemical Corporation) and 50 μmol/L CaCl_2_ in perfusion buffer). Once the heart became slightly pale and flaccid, the heart was transferred into cell culture dishes containing digestion buffer. The digested tissue was gently teased into small pieces with two fine‐tip forceps to release the cells. The cells were centrifuged for 5 minutes at 400 *g*, fixed with 4% paraformaldehyde (Sigma) for 10 minutes and permeablized with 0.2% Triton X‐100 in 10% FBS for 30 minutes. Cell death was detected with TUNEL Assay Kit—BrdU‐Red (Abcam, Cat. No. ab66110) according to the instructions. Cells were analysed with flow cytometry. And the cell death rate of transplanted cells was assessed by comparing to GFP^+^TUNEL^+^ to total GFP+ cells.

### 2.9 Statistical analysis

Data were analysed with SPSS software (SPSS Inc). Student *t* test was used for two group comparison and 1‐way ANOVA for multiple group comparison with normal data distribution, parametric test and Turkey post hoc tests. A level of *P* < .05 was considered statistically significant.

## 3 RESULTS

So far, the mouse fibroblast cells have been successfully converted into cardiac progenitor‐like cells by using retrovirus or lentivirus approaches.^9^, ^10^, ^11^, ^12^ However, their genome integration characteristic significantly hampered their therapeutic applications, especially for human induced cardiac progenitor cell generation and their potential clinical applications in the future. To eliminate their potential tumorigenesis risks, four well established transgene‐free approaches for somatic reprogramming, including episome,^20^ minicircle,^21^ self‐replicative RNA ^22^ and sendai virus,^23^ were applied into iCS generation, one type of induced cardiac progenitor cells.^10^

**FIGURE 1:**
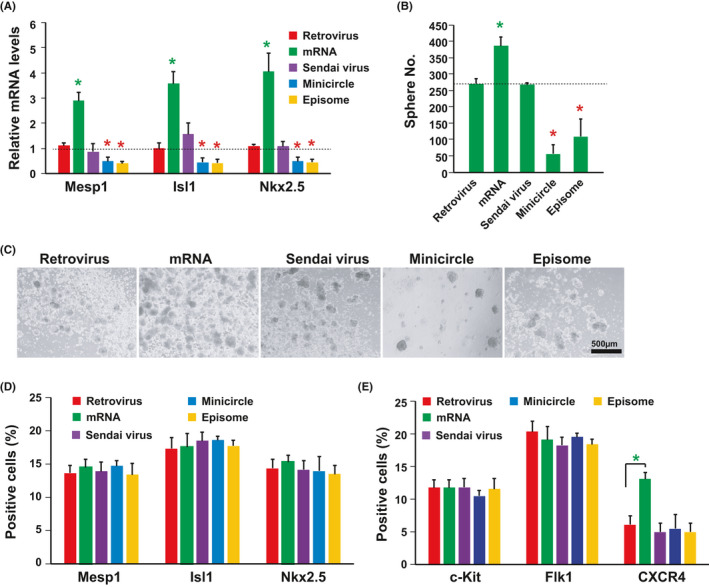
Generation of iCS with transgene‐free approaches. A, qPCR analysis of Mesp1, Isl1 and Nkx2.5 showed that their mRNA levels were upregulated by SKO (Sox2, Klf4 and Oct4) overexpressed with retrovirus, synthetic self‐replicative RNA, sendai virus, minicircle or episome approach at day 18 of somatic reprogramming (n = 3, biological replicates; *P < .05, transgene‐free group vs retrovirus group). B, Sphere number counting. The iCS was formed 2 d after plating on poly‐D‐Lysin coated plates after 18 d of reprogramming (n = 3, biological replicates; *P < .05, transgene‐free group vs retrovirus group). C, Representative figures of iCS generated with transgene‐free approaches. D, Percentage of Mesp1, Isl1 and Nkx2.5 positive cells within iCS via immunostaining (n = 3) at day 2 after shere formation. E, Percentage of c‐Kit, Flk1 and CXCR4 positive cells within iCS analysed with flow cytometry (n = 3). Student *t* test was used for assessing the significances between different transgene‐free approaches and retrovirus approach. A level of *P* < .05 was considered statistically significant

After 18 days (starting from SKO infection/transfection) of reprogramming, the mRNA levels of cardiac progenitor marker Mesp1, Isl1 and Nkx2.5 were significantly upregulated in the group with mRNA approach when comparing with the retrovirus approach (Figure 1A). In the contrast, their mRNA levels decreased in the groups with episome and minicircle approaches, while they showed similar levels between the sendai virus and retrovirus approach (Figure 1A). Sphere counting after plating onto poly‐d‐lysine coated plates showed similar pattern to the gene expression of cardiac progenitor markers (Figure 1B,C). Furthermore, the iCS generated with all five approaches contained similar levels of Mesp1, Isl1 and Nkx2.5 positive cardiac progenitor cells (Figure 1D). And they also had similar levels of stem cell marker c‐Kit and Flk1 positive cells (Figure 1E). However, the iCS generated with mRNA approach had more CXCR4 positive cells (Figure 1E). As a result, the mRNA approach was better than the other four approaches. Because the mRNA approach produced more iCS and CXCR4 positive cells with higher mRNA level of cardiac progenitor markers. And the exogenous RNAs were undetectable in the iCS cells (FigureS 1). Thus, the iCS generated with mRNA approach was subjected to subsequent studies.

**FIGURE 2:**
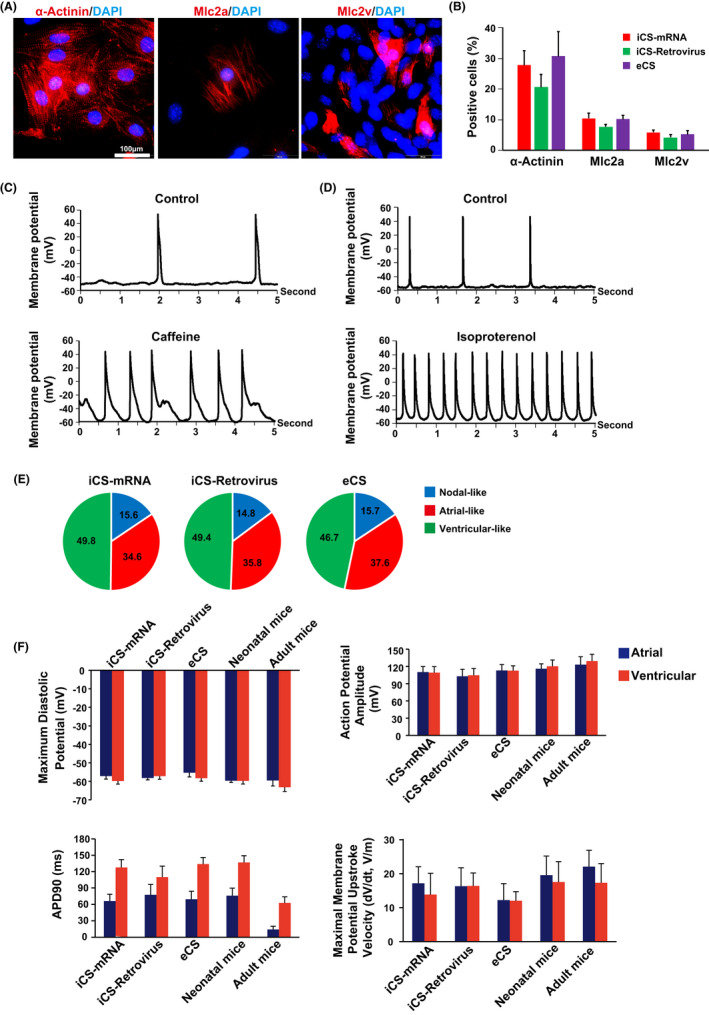
Functional characterizations of iCS generated with synthetic self‐replicative mRNA approach. A, iCS generated with synthetic self‐replicative mRNA approach differentiated into α‐Actinin, Mlc2a and Mlc2v positive cardiomyoctes. B, iCS generated with synthetic self‐replicative mRNA approach had a similar cardiomyocyte differentiation capability with iCS generated with retrovirus and eCS, shown by measuring α‐Actinin, Mlc2a and Mlc2v positive cell via flow cytometry analysis (n = 3); (C) Whole‐cell patch clamp analysis of cardiomyocytes, which were differentiated from iCS generated with synthetic self‐replicative mRNA approach, treated with or without 10 mmol/L caffeine. D, Whole‐cell patch clamp analysis of cardiomyocytes, which were differentiated from iCS generated with synthetic self‐replicative mRNA approach, treated with or without 10 μmol/L beta‐adrenergic agonist isoproterenol. E, iCS generated with retrovirus (n = 54) or synthetic self‐replicative mRNA (n = 52) approach and eCS (n = 51) had a similar capability to differentiate into nodal, atrial and ventricular‐like cardiomyocytes as determined by resting membrane potential and action potential (AP) morphology measured by whole‐cell patch clamp. F, Cardiomyocytes differentiated from iCS generated with retrovirus (n = 46) or synthetic self‐replicative mRNA (n = 44) approach had similar electrophysiological properties to eCS (n = 43) and neonatal cardiomyocytes from the perspective of maximum diastolic potential, action potential amplitude, action potential duration at 90% repolarization (APD90) and maximal membrane potential upstroke velocity via patch clamp (n = 20 for neonatal and adult cardiomyocytes). Mlc2a: myosin light chain 2 atrial; Mlc2v: myosin light chain 2 ventricular; eCS: endogenous cardiosphere isolated from mice. One‐way ANOVA with normal data distribution, parametric test and Turkey post hoc tests was for assessing the significances among groups. A level of *P* < .05 was considered statistically significant

**FIGURE 3:**
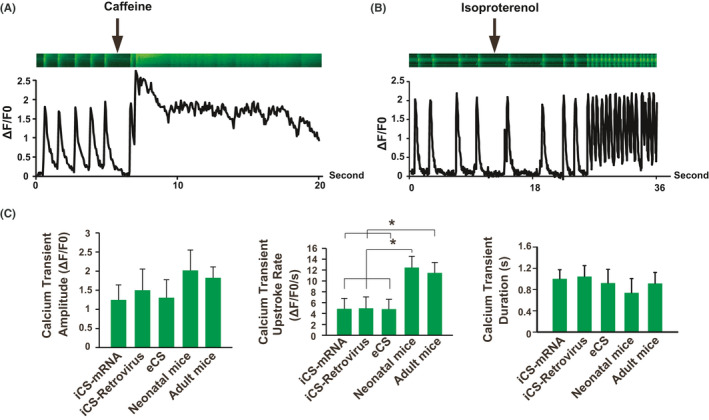
Calcium handling characterizations of iCS generated with synthetic self‐replicative mRNA approach. A, Calcium transient analysis of cardiomyocytes, which were differentiated from iCS generated with synthetic self‐replicative mRNA approach, treated with or without 10 mmol/L caffeine. B, Calcium transient analysis of cardiomyocytes, which were differentiated from iCS generated with synthetic self‐replicative mRNA approach, treated with or without 10 μmol/L beta‐adrenergic agonist Isoproterenol. C, Cardiomyocytes differentiated from iCS generated with retrovirus (n = 10) or synthetic self‐replicative mRNA (n = 10) approach had similar calcium handling properties to eCS‐derived cardiomyocytes (n = 10), *P < .05 as indicated with arrows. eCS: endogenous cardiosphere isolated from mice. One‐way ANOVA with normal data distribution, parametric test and Turkey post hoc tests was for assessing the significances among groups. A level of *P* < .05 was considered statistically significant

After 15 days (starting from cardiac differentiation) of cardiomyocytes differentiation, the iCS generated with mRNA approach could form beating clusters (Movie S1) and express different cardiomyocytes markers including α‐Actinin, Mlc2a (myosin light chain 2 atrial, atrial specific marker) and Mlc2v (myosin light chain 2 ventricular, ventricular specific marker) (Figure 2A). The cardiomyocytes differentiation efficiencies were comparable among iCS generated with mRNA and retrovirus approaches, and the isolated endogenous cardiosphere (eCS) from the mouse heart (Figure 2B). Patch clamp analysis of the differentiated cardiomyocytes from iCS generated with mRNA approach showed that caffeine could stimulate extra calcium release and prolong the action potential duration (Figure 2C). Furthermore, beta‐adrenergic agonist isoproterenol increased the spontaneous action potential rate (Figure 2D). Thus, these differentiated cardiomyocytes could functionally response to caffeine and isoproterenol stimulation. Then, the nodal‐, atrial‐ and ventricular‐like cardiomyocytes were defined by the ratio of action potential duration (APD) at 90%‐50% repolarization.^10^, ^26^ Data showed that the iCS generated with mRNA and retrovirus approaches had similar levels of differentiated nodal‐, atrial‐ and ventricular‐like cardiomyocytes as eCS (Figure 2E). Further functional comparison among cardiomyocytes derived from iCS generated with mRNA and retrovirus approaches, eCS and freshly isolated mouse native cardiomyocytes (neonatal and adult cardiomyocytes) showed that they had similar maximum diastolic potential, action potential amplitude, APD at 90% repolarization and the maximal membrane potential upstroke velocity (Figure 2F). However, the adult cardiomyocytes had a significantly shorter APD at 90% repolarization compared with other cardiomyocytes (Figure 2F). Calcium transient analysis further confirmed that the differentiated cardiomyocytes from iCS generated with mRNA approach could functionally response to caffeine and isoproterenol stimulation (Figure 3A,B). Furthermore, the iCS generated with mRNA or retrovirus approach, and eCS derived cardiomyocytes had similar level of calcium transient amplitude and duration (Figure 3C). However, these differentiated cardiomyocytes had a significantly lower calcium transient upstroke rate than native cardiomyocytes (neonatal and adult cardiomyocytes) (Figure 3C). In summary, our data showed that the in vitro functional parameters of cardiomyocytes derived from iCS generated with mRNA or retrovirus approach and eCS were similar; but were less mature compared with native cardiomyocytes. Given the mRNA approach produced more iCS with higher percentage of CXCR4 positive stem cells, the iCS generation with mRNA approach is better than the previous retrovirus approach.^10^

**FIGURE 4:**
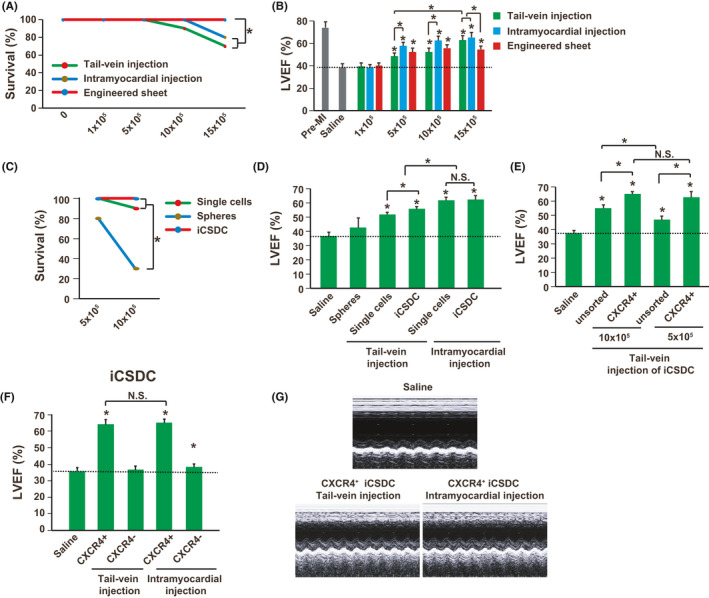
Transplantation of iCS generated with synthetic self‐replicative mRNA approach following MI. A, Survival of mice transplanted with different cell number of iCS through different delivery approaches at week 4 after MI induction and iCS transplantation (n = 20 for each group). B, Assessment of LVEF by echocardiogram at week 4 after cell transplantation (n = 8). C, Survival of mice transplanted with different cell number of iCS in the form of sphere or single cells through tail‐vein injection at week 4 after MI induction and cell transplantation (n = 30 for each group). D, Assessment of LVEF by echocardiogram at week 4 after cell transplantation (n = 8). E, Assessment of LVEF by echocardiogram at week 4 after transplanting sorted CXCR4^+^ or unsorted iCSDC with different cell number via tail‐vein injection (n = 8). F, Assessment of LVEF by echocardiogram at week 4 after CXCR4^+^ or CXCR4^‐^ iCSDC transplantation via tail‐vein or intramyocardial injection (n = 8). G, Representative M‐mode echocardiographic tracing at week 4 after MI induction and cell transplantation. LVEF: left ventricular ejection fraction; iCSDC: iCS derived single cells after 3 passages; NS indicates no significant difference; *P < .05. One‐way ANOVA with normal data distribution, parametric test and Turkey post hoc tests was for assessing the significances among groups. A level of *P* < .05 was considered statistically significant

**FIGURE 5:**
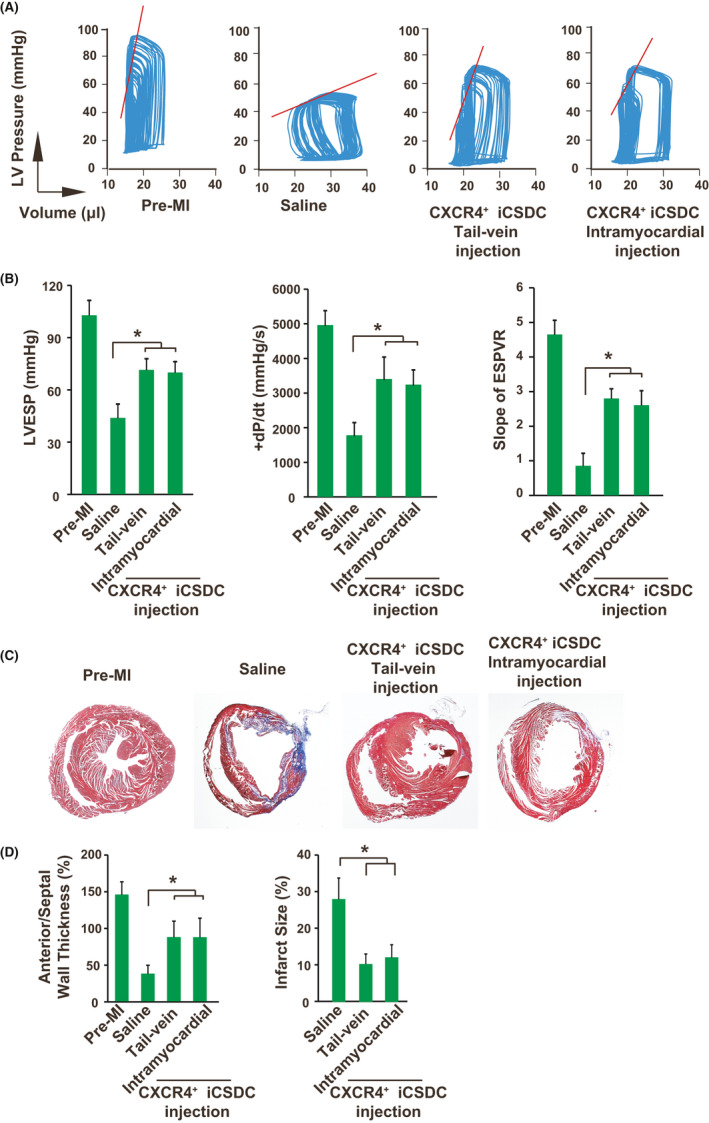
Transplantation of CXCR4^+^ iCSDC following MI. A, Representative tracing of the pressure‐volume loop assessment to measure slope of end‐systolic pressure‐volume relationship (ESPVR) at week 4 after cell transplantation showing cardiac function improvement. B, Hemodynamic assessment at week 4 after cell transplantation showed both tail‐vein and intramyocardial delivery of CXCR4^+^ iCSDC achieved a similar level of cardiac function improvement (n = 8). C, Trichrome staining of paraffin‐embedded heart sections at week 4 after cell transplantation indicated wall thickness increase and infarct size decrease. D, Left panel: left ventricular wall thickness was measured by the ratio of anterior to septal wall thickness at week 4 after cell transplantation (n = 8); Right panel: infarct size measurement by the percentage area with collagen deposit using trichrome staining at week 4 after cell transplantation (n = 8). +dP/dt, peak rate of pressure rise; ESPVR, end‐systolic pressure‐volume relationship; iCSDC, iCS derived single cells after 3 passages; LVESP, left ventricular end‐systolic pressure; Pre‐MI, before myocardial infarction; *P < .05. One‐way ANOVA with normal data distribution, parametric test and Turkey post hoc tests was for assessing the significances among groups. A level of *P* < .05 was considered statistically significant

**FIGURE 6:**
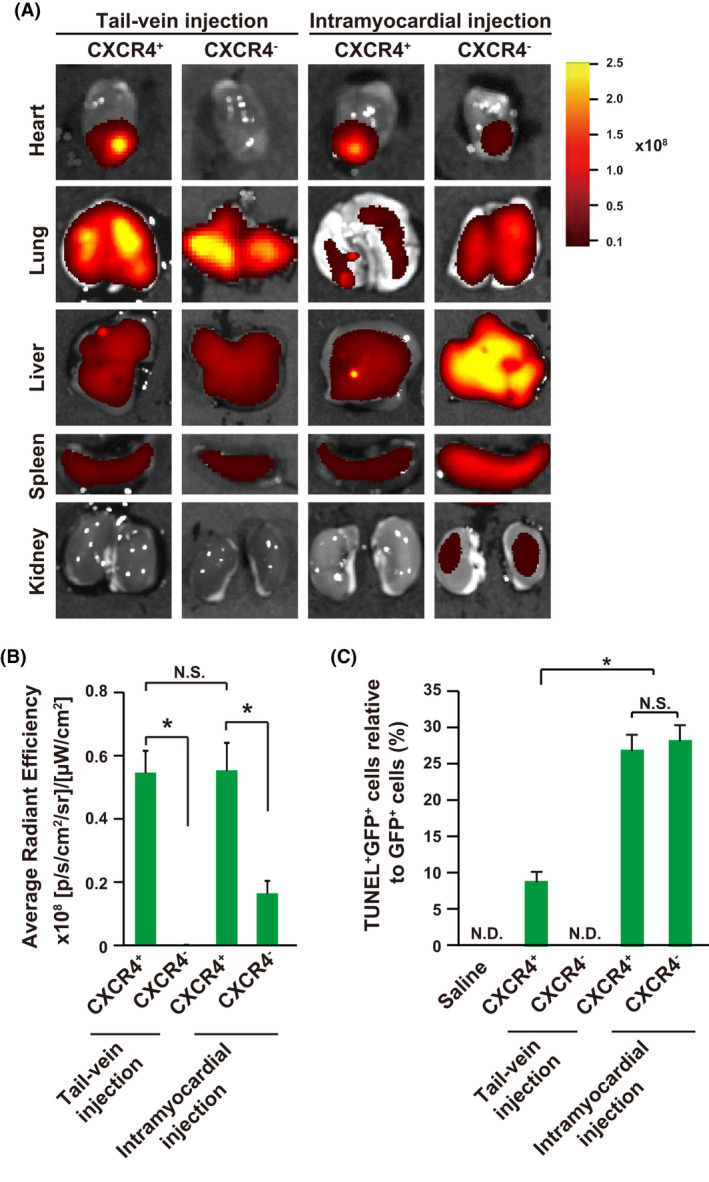
Cardiac recruitment of CXCR4^+^ iCSDC. A, Representative figures of DiR labelled cells in the heart, lung, liver, spleen and kidney at week 4 after cell transplantation in a mouse model of myocardial infarction, heatmap showed the average radiant efficiency. B, Average radiant efficiency of DiR labelled cells in the heart after CXCR4^+^ iCSDC transplantation indicated both tail‐vein and intramyocardial delivery achieved similar engraftment (n = 8). C, The cell death rate of transplanted cells in the heart was assessed by comparing to GFP^+^TUNEL^+^ to total GFP+ cells, analysed by flow cytometry. *P < .05; NS indicates no significant difference; ND indicates not detected. One‐way ANOVA with normal data distribution, parametric test and Turkey post hoc tests was for assessing the significances among groups. A level of *P* < .05 was considered statistically significant

Although it has been demonstrated that the intramyocardial injection retained more transplanted cells in the heart, this delivery strategy showed less consistency and would induce extra injuries to the heart.^13^ Thus, alternative delivery strategies should be optimized to improve the cardiac engraftment efficiency. Three different transplantation strategies were compared, including tail‐vein injection, intramyocardial injection and engineered sheet transplantation. For iCS transplantation, high cell number delivery, especially 15 × 10^5^ cells per mouse (18 ± 1g), with tail‐vein or intramyocardial injection induced significantly mouse death, while engineered sheet transplantation did not (Figure 4A). As expected, intramyocardial delivery showed improved left ventricular ejection fraction (LVEF) than tail‐vein delivery at week 4 after cell transplantation as determined by echocardiogram (Figure 4B). Interestingly, high cell number transplantation (15 × 10^5^ vs 5 × 10^5^ cells) via tai‐vein injection had enhanced cardiac function improvement while intramyocardial and engineered sheet transplantation did not (Figure 4B). Furthermore, tai‐vein delivery with high cell number (15 × 10^5^ cells) showed comparable cardiac function improvement with intramyocardial delivery, indicating that further optimization of tail‐vein delivery is possible to reach comparable therapeutic effects with intramyocardial delivery. It has been demonstrated that transplanting the cardiospheres in the form of the sphere was more effective than the cardiosphere‐derived cells, as the three‐dimensional structure maintained the niche for stem cells.^27^ Furthermore, our previous study showed that continuous cell passaging would increase the c‐Kit, Flk1 and CXCR4 positive cells within the iCS.^10^ Thus, the mouse survival and cardiac function improvement were compared among iCS spheres, iCSDC (iCS derived cells after 3 passages) and single cells directly dissociated from iCS delivered via tail‐vein injection. Data showed that sphere delivery via tail‐vein injection further reduced mouse survival, possible resulting from vein blockage induced by large size of sphere (Figure 4C). Interestingly, iCSDC had better cardiac function improvement than iCS derived single cells (10 × 10^5^ cells per mouse) (Figure 4D). Therefore, the iCSDC was used for subsequent experiments. Another limitation of tail‐vein injection with high cell number is the reduced mouse survival (Figure 4A). Thus, purification of effective subpopulation of iCS is the possible way to reduce cell number for transplantation while maintaining their therapeutic efficacy. It has been demonstrated that the CXCR4^+^ stem cells had better cardiac engraftment after MI and showed improved therapeutic effects.^15^, ^16^, ^17^, ^18^, ^19^ And our previous study showed that the CXCR4^+^ cells had the capability to differentiate into cardiomyocytes but not those negative cells.^10^ Moreover, the CXCR4^+^ subpopulation cells were further enriched within in the iCS on further passages.^10^ And our data here showed that the iCS generated with mRNA approach contained more CXCR4^+^ cells than retrovirus approach which was used previously (Figure 1E). Indeed, when transplanting the same cell number of iCSDC, the purified CXCR4^+^ iCSDC had better cardiac function improvement than the unpurified iCSDC (Figure 4E). Furthermore, transplanting less cell number of CXCR4^+^ iCSDC (5 × 10^5^ cells per mouse) showed similar cardiac function improvement with high cell number transplantation (10x10^5^ cells per mouse) (Figure 4E). In addition, CXCR4^+^ iCSDC delivered via tail‐vein injection efficiently improved cardiac function, with similar levels with intramyocardial injection (Figure 4E‐G). Invasive hemodynamic assessment at week 4 also confirmed that compared with saline, both tail‐vein and intramyocardial transplantation of CXCR4^+^ iCSDC (5 × 10^5^ cells per mouse) significantly improved left ventricular end‐systolic pressure (LVESP), maximum increase in left ventricular pressure (+dP/dt) and the slope of end‐systolic pressure‐volume relationship (ESPVR) (Figure 5A,B). Histological examination with Trichrome staining showed that the CXCR4^+^ iCSDC had similar therapeutic effects between tail‐vein and intramyocardial delivery, from the perspectives of increasing anterior/septal ventricular wall thickness and reducing infarct size (Figure 5C,D). Therefore, the CXCR4^+^ iCSDC transplantation via tail‐vein delivery might be a better therapeutic approach than intramyocardial transplantation with comparable therapeutic effects. Because the CXCL12/CXCR4 pathway is responsible for the cardiac recovery after MI and recruiting CXCR4 positive cells into injured heart.^15^, ^28^ The cardiac engraftment of transplanted iCSDC was assessed by in vivo tracking with using DiR labelling. Four weeks after iCSDC transplantation, the CXCR4^+^ iCSDC was engrafted or recruited to the heart at similar levels between tail‐vein and intramyocardial injection (Figure 6A,B). However, the CXCR4^+^ iCSDC delivered with tail‐vein injection had lower cell death rate in the heart when comparing to intramyocardial injection (Figure 6C). Therefore, transplanting CXCR4^+^ iCSDC via tail‐vein injection has comparable cardiac retention with direct intramyocardial injection.

## 4 DISCUSSIONS

Although the cardiac progenitor cell therapy had been proposed as a promising approach for cardiac regeneration and MI treatment,^5^, ^6^ it has been noticed recently that the endogenous cardiac progenitor cells might not exist in the adult heart.^7^, ^8^ Therefore, cardiac progenitor‐like cells generated through somatic reprogramming represents a promising strategy to treat MI.

Previously, we and others had successfully reprogrammed mouse fibroblast cells into cardiac progenitor‐like cells via somatic reprogramming, including induced cardiosphere (iCS), Nkx2.5^+^ and Flk1^+^PdgfR⍺^+^ induced cardiac progenitor cell (iCPC).^9^, ^10^, ^11^, ^12^ However, the genome integration characteristic of retrovirus/lentivirus hampered their therapeutic applications due to the risk of tumour formation. Thus, we screened several transgene‐free approaches for somatic reprogramming, including episome,^20^ minicircle,^21^ self‐replicative RNA^22^ and sendai virus.^23^ And we found that the self‐replicative RNA approach produced more iCS, which had cardiomyocyte differentiation ability and therapeutic effects on the mouse model of MI. And they were comparable with endogenous cardiospheres and iCS generated with retrovirus. Self‐replicative RNA vector still contains virus RNAs which would trigger immune responses and might reduce the cell retention ratio resulting from immune rejection. Thus, other optimized strategies are also needed in the future.

Another issue is that the intramyocardial injection, which is widely used in most studies, would introduce extra injuries to the heart, although this approach shows better engraftment and therapeutic effects in the animal models.^10^, ^13^ Intravenous injection is another delivery approach for stem cell therapy. However, as there are limited cells homing to the cardiac tissue, this is not effective as the intramyocardial injection for treating MI. CXCL12‐CXCR4 pathway contributes to tissue regeneration via recruiting stem cells to the injured sites,^14^ and thus has been applied to improve the efficacy of stem cell therapy on MI.^15^, ^16^, ^17^, ^18^, ^19^ Indeed, our data here showed that the CXCR4^+^ subpopulation of iCS derived cells (iCSDC) delivered by intravenous injection had similar therapeutic effects with intramyocardial injection on the mouse model of MI, representing a safer delivery approach.

In conclusion, the mouse iCS could be generated by synthetic self‐replicative RNAs, a transgene‐free approach, with enhanced efficiency. The CXCR4^+^ subpopulation of iCSDC delivered by intravenous injection was found to have similar therapeutic effects with intramyocardial injection on the mouse model of MI, representing a safer delivery approach. Thus, the optimized strategy for iCS generation is safer and has more therapeutic potentials.

## CONFLICT OF INTEREST

The authors declare no commercial or financial conflict of interest.

## AUTHOR CONTRIBUTIONS

JX designed and supervised the project, interpreted the data, and wrote the manuscript. HW and XW conducted the cell culture experiments. ZM and JY conducted the animal studies. JC, LY and XZ analysed the data. LL and ZH analysed the data and corrected the manuscript.

## ETHICAL APPROVAL

This study was approved by the ethics committee of Shenzhen University and followed the tenants of the Declaration of Helsinki.

## Supporting information

Figure S1

Supplementary Material

## Data Availability

All related data are available under request.
